# Enhancer of zeste homolog 2 is involved in the proliferation of endometrial carcinoma

**DOI:** 10.3892/ol.2014.2437

**Published:** 2014-08-12

**Authors:** NAN JIA, QING LI, XIANG TAO, JIEYU WANG, KEQIN HUA, WEIWEI FENG

**Affiliations:** 1 Department of Gynecology, Obstetrics and Gynecology Hospital, Fudan University, Shanghai 200090, P.R. China; 2 Shanghai Key Laboratory of Female Reproductive Endocrine-Related Diseases, Obstetrics and Gynecology Hospital, Fudan University, Shanghai 200090, P.R. China; 3 Department of Pathology, Obstetrics and Gynecology Hospital, Fudan University, Shanghai 200090, P.R. China

**Keywords:** endometrial carcinoma, EZH2, cell proliferation, endometrial hyperplasia

## Abstract

Endometrial carcinoma is the second most common gynecological malignancy of the female genital tract worldwide. Enhancer of zeste homolog 2 (EZH2), a critical component of the polycomb repressive complex 2, has been found to be involved in multiple biological processes and is overexpressed in several types of cancer. Previous studies have demonstrated that EZH2 is associated with endometrial carcinoma. The present study showed that EZH2 was overexpressed in complex hyperplasia, atypical hyperplasia and endometrial cancer, but not in simple hyperplasia and normal endometrium. Additionally, by analyzing the correlation between EZH2 expression and clinicopathological characteristics, the expression of EZH2 was found to be associated with myometrial invasion and lymph-vascular space invasion of endometrial cancer. Furthermore, small interfering RNA was utilized to investigate the role of EZH2 in endometrial carcinoma cell proliferation, and the results showed that EZH2 knockdown suppressed the proliferation of endometrial carcinoma cells *in vitro*. Therefore, these findings indicate that EZH2 expression may predict a more aggressive biological behavior in endometrial carcinoma and it may provide potential therapeutic targets for treatment of endometrial carcinoma.

## Introduction

Endometrial carcinoma (EC) is the second most common gynecological malignancy of the female genital tract worldwide (1). In the United States, with 47,130 new cases and 8,010 mortalities projected in 2012 (2), the majority of women (80–85%) present with early-stage disease, and surgery in the form of hysterectomy and bilateral salpingo-oophorectomy is curative. However, a proportion of the cases present with advanced disease or develop disease recurrence or metastasis, which are associated with poor survival (3).

Histologically, endometrial cancer can be divided into two types (4). The common and estrogen-dependent type is called type I cancer, and is generally diagnosed at an early stage and as a result has an improved prognosis. However, certain cases with advanced stage and high tumor grade have a poor survival. Type II cancer, which usually has a papillary serous or clear cell pattern, is likely associated with p53 mutation. The probability of surviving 5 years with this type of cancer is considerably lower than that for the type I form, even with early stage diseases (5). Therefore, there is an urgent requirement for the identification of new therapeutic targets.

Enhancer of zeste homolog 2 (EZH2), a critical component of the polycomb repressive complex 2, has intrinsic histone methyl transferase activity that mediates gene silencing by catalyzing trimethylation on lysine 27 of histone H3 (6). EZH2 has been found to be involved in multiple biological processes, such as tumor proliferation (7), cell cycle, senescence (8), metastasis and angiogenesis (9). EZH2 is overexpressed in aggressive forms of prostate (10), breast (11) and bladder (12) cancer.

Nevertheless, EZH2 expression is associated with a high proliferation rate and aggressive tumor subgroups of endometrial cancer (13). EZH2 expression has been found to be positively associated with lipocalin 2 expression, which is associated with aggressive features of endometrial cancer (14). Inhibition of EZH2 expression is associated with decreased tumor cell proliferation, migration and invasion in endometrial cancer cell lines, which is parallel to an increased expression of Wnt pathway inhibitors, sFRP1 and DKK3, and a concomitant decrease in β-catenin levels (15).

However, the role of EZH2 in endometrial cancer has not been fully determined. In the present study, the expression of EZH2 in endometrial cancer and precancerous lesions was evaluated, and the potential role of EZH2 in endometrial cancer cell proliferation was further investigated.

## Materials and methods

### Cell culture

Human endometrial carcinoma cell lines, Hec-1a and Ishikawa (provided by Dr Yinhua Yu; Anderson Cancer Center, Houston, TX, USA), were maintained in McCoy’s 5A medium (Jinuo Co., Ltd, Shanghai, China) supplemented with 10% fetal bovine serum (Gibco-BRL, Rockville, IN, USA). The cells were incubated at 37°C in 5% CO_2_.

### Tissue samples

A total of 92 endometrial tissues (including 24 normal endometrium, 14 simple hyperplasia, 6 complex hyperplasia, 15 atypical hyperplasia and 33 endometrial cancer samples) were obtained from patients who underwent surgery between August 2008 and December 2012 at the Obstetrics and Gynecology Hospital, Fudan University (Shanghai, China). Normal endometrium and simple and complex hyperplasia samples were from patients who received dilation and curettage, whereas atypical hyperplasia and cancer tissues were from patients who received hysterectomy. All specimens were reviewed by an experienced pathologist. The patients’ demographic profiles and the pathology files were tabulated. For endometrial cancer patients, the clinicopathological factors, such as age, tumor grade, depth of myometrial invasion, lymph-vascular space invasion (LVSI) and nodal metastasis, were analyzed. The surgical pathology stage was determined by the 1998 International Federation of Gynecology and Obstetrics (FIGO) guidelines (16). All patients provided written informed consent permitting the use of their tissue for research at the time specimens were collected. This study was approved by the institutional review board of the Obstetrics and Gynecology Hospital, Fudan University.

### Immunohistochemistry

Immunohistochemistry (IHC), antigen retrieval and antibody dilution were optimized prior to the study onset. All endometrium specimens were reviewed by experienced pathologists to confirm the diagnosis. To ensure uniformity, all sections were processed simultaneously. Four-micrometer paraffin sections adjacent to the hematoxylin and eosin sections used for histological assessment were mounted onto Superfrost Plus slides (Menzel, Braunschweig, Germany). Slides were subjected to immunoperoxidase staining for EZH2 (5246S; Cell Signaling Technology, Inc., Danvers, MA, USA). Endogenous peroxidase activity was blocked using 0.3% hydrogen peroxide. Antigen retrieval was performed by heating the sections for 30 min in a microwave oven with 10 mM sodium citrate (pH 6.0). The slides were then incubated with monoclonal antibodies against EZH2 (1:50; rabbit anti-human, -rat, -mouse and -monkey; Cell Signaling Technology, Inc.) at 4°C overnight. Slides were washed and incubated with the biotinylated secondary antibody (polyclonal goat anti-rabbit; Histostain-Plus IHC kit; Mingrui Biotech, Shanghai, China) for 45 min at 37°C and washed with phosphate-buffered saline. Slides were incubated with avidin-biotin-peroxidase (Histostain-Plus IHC kit; Mingrui Biotech) for 10 min at room temperature and incubated with diaminobenzidine (Mingrui Biotech) for 2 min. Finally, slides were counterstained with hematoxylin and evaluated at a magnification of ×200 using light microscopy. The intensity of positive cells was graded from 0 to 3 (0, negative; 1, weak; 2, medium; 3, strong). The scores were determined independently by two observers, and the average of their scores was used for evaluation. For estrogen receptor (ER) (1:150), progesterone receptor (PR) (1:160), p53 (1:500) and Ki-67(1:200) (Dako, Glostrup, Denmark) detection, antigens were unmasked by treating the slides with Target Retrieval Solution, High pH (Dako) for 30 min at 95°C. High-grade serous adenocarcinoma of the ovary was used as positive control for p53 and Ki-67, while ductal carcinoma of the breast was used as positive control for ER and PR. Expression of ER and PR was considered as positive when >1% of the nuclei of cells in the epithelium showed immunoreactivity. Expression of p53 was considered as positive when immunohistochemical staining was observed in >5% of the nuclei of cells in the epithelium. The percentage of the nuclei staining of Ki-67 was determined independently by two observers.

### Western blot analysis

Cells were lysed in RIPA buffer (150 mM NaCl, 50 mM Tris-base, 5 mM EDTA, 1% NP-40, 0.25% deoxycholate, pH 7.4). Protein concentrations were measured by the BCA protein assay (23227; Thermo Fisher Scientific, Waltham, MA, USA). Equal amounts of protein were resolved by SDS-PAGE, transferred to PVDF membranes and incubated with appropriate primary antibodies (monoclonal rabbit anti-human EZH2 antibody, 5246S, 1:1000, Cell Signaling Technology, Inc.; monoclonal rabbit anti-human GAPDH antibody, 5632–1, 1:5000, Epitomics, Burlingame, CA, USA). Immune complexes were detected with a goat anti-rabbit horseradish peroxidase-conjugated secondary antibody (1:5000; SSA005; Sino Biological Inc., Beijing, China) and enhanced chemiluminescence reagent (32109; Thermo Fisher Scientific).

### EZH2 siRNA transfection

Hec-1a and Ishikawa cells were plated on six-well plates at a density of 2×10^5^ cells/well and grown overnight until 30–40% confluency. The cells were transfected with validated siRNA for EZH2 (sense: 5′-GUGUAUGAGUUUAGAGUCATT-3′) and a scramble siRNA-FAM (negative control) (synthesized by Jima, Co., Ltd, Shanghai, China) at a concentration of 100 nM. using Lipofectamine 2000 transfection reagent (Invitrogen Life Technologies, Carlsbad, CA, USA) according to the manufacturer’s instructions. The medium was replaced with standard culture medium 12 h post-transfection. Transfection was repeated 72 h after the first transfection.

### In vitro cell proliferation assay

Cell proliferation was assayed using a cell proliferation kit, Cell Counting Kit-8 (CCK-8; Dojindo Molecular technologies, Inc., Kyushu, Japan) according to the manufacturer’s instructions. Hec-1a and Ishikawa cells were seeded in sextuplicate onto 96-well tissue culture plates at a density of 2×10^3^ cells/well the day before EZH2 siRNA transfection. Cell growth was analyzed at a wavelength of 450 nm at 0, 48, 72, 96 and 120 h after transfection using Multiskan MK3 (Thermo Fisher Scientific). Experiments were performed in triplicate.

### Statistical analysis

The data are presented as the means ± standard deviation. Statistical analyses were performed using SPSS 15.0 software (SPSS, Inc., Chicago, IL, USA). Two independent samples non-parametric tests were utilized to analyze the immunohistochemistry results. Fisher’s exact tests were utilized to analyze the association between EZH2 methylation levels and clinicopathological characteristics. Paired-sample t-tests were utilized to analyze the CCK-8 results of EZH2 knockdown. P<0.05 was considered to indicate a statistically significant difference.

## Results

### EZH2 is overexpressed in complex hyperplasia, atypical hyperplasia and endometrial carcinoma

**Figure 1:**
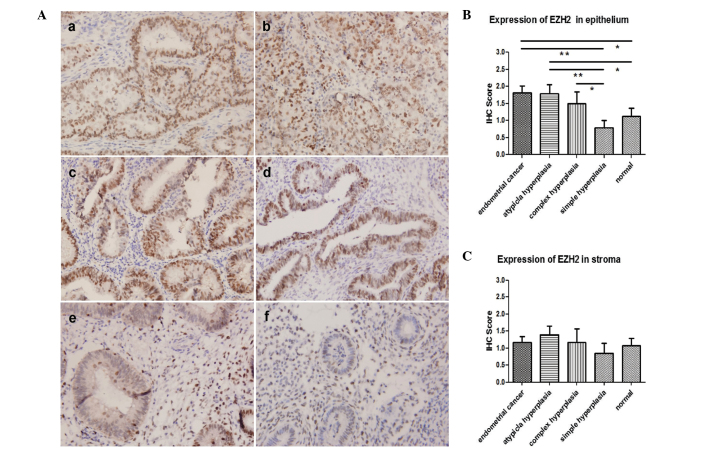
Expression of EZH2 in endometrial tissues. (A) EZH2 is overexpressed in the epithelium of endometrial cancer, atypical hyperplasia and complex hyperplasia compared with that in simple hyperplasia and normal endometrium. a, type 1 endometrioid endometrial cancer; b, type 2 endometrial cancer; c, atypical hyperplasia; d, complex hyperplasia; e, simple hyperplasia; f, proliferative endometrium. All images were captured at ×200 magnification using light microscopy and stained with hematoxylin. (B) Expression of EZH2 is higher in the epithelium of endometrial cancer, atypical hyperplasia and complex hyperplasia than in simple hyperplasia and normal endometrium. (C) Expression of EZH2 showed no difference in the stroma among groups. ^*^P<0.05 and ^**^P<0.01. EZH2, enhancer of zeste homolog 2.

Immunohistochemistry was performed in 92 endometrium tissues, including 24 normal endometrium (21 proliferative endometrium, two secretory endometrium and one atrophic endometrium; age range, 38–66), 14 simple hyperplasia (age range, 30–76), six complex hyperplasia (age range, 44–62), 15 atypical hyperplasia (age range, 36–64) and 33 endometrial cancers (27 type I and six type II; age range, 37–78). The age distribution was similar among groups (P>0.05). Overexpression of EZH2 was observed in the epithelium of endometrial cancer, atypical hyperplasia and complex hyperplasia compared with the expression in simple hyperplasia and normal endometrium. No difference was seen among endometrial cancer, atypical hyperplasia and complex hyperplasia, or between simple hyperplasia and normal endometrium, in terms of epithelial EZH2 expression (Fig. 1A and B). Expression of EZH2 showed no difference in the stroma among all groups (Fig. 1C).

### Expression of EZH2 is associated with myometrial invasion of endometrial cancer

The association between expression of EZH2 and clinicopathological characteristics of 33 endometrial cancer tissues was analyzed. The samples were grouped based on whether they had high (IHC score, 2 or 3) or low (IHC score, 0 or 1) expression of EZH2 (Table I). Tumor grade; FIGO stage; depth of myometrial invasion; LVSI; nodal metastasis status; ER, PR and p53 expression and Ki-67 labeling index of the tumor were determined, and patients ranged in age from 37 to 78 years (medium age, 55 years). High expression of EZH2 was observed in 64% of these cases. As shown in Table I, high EZH2 expression was associated with deep myometrial invasion. In samples of low EZH2 expression, two cases (17%) were limited to the endometrium, 10 (83%) cases occupied less than half of the myometrium and none occupied more than half of the myometrium. However, in the high EZH2 expression group, 14 cases (67%) were found to occupy less than half of the myometrium, while seven (33%) cases demonstrated deep (≥1/2) myometrium infiltration (P=0.013). Cases with deeper myometrial invasion were more likely to be EZH2-overexpressing.

In addition, high EZH2 expression appeared to be associated with the presence of LVSI. Although the P-value (0.065) was not significant, this may have been due to the small sample size. No correlation was noted between EZH2 expression and other clinicopathological characteristics.

### EZH2 is involved in cell proliferation of endometrial carcinoma

**Figure 2:**
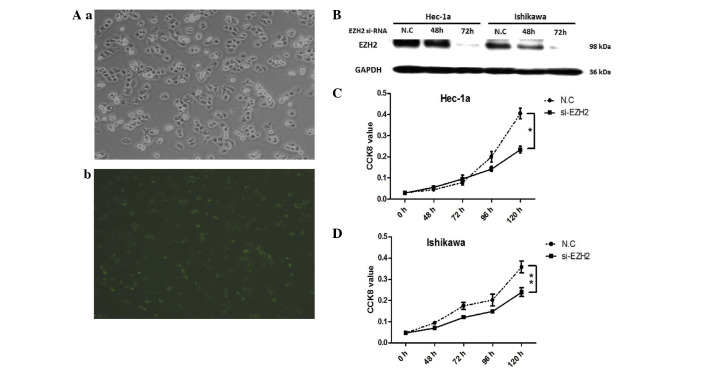
EZH2 knockdown inhibits the growth of endometrial cancer cells. (A) FAM-labeled scramble siRNA was transfected into Hec-1a and Ishikawa cells and the transfection efficiency was >90%; a, bright field of the cultured Hec-1a cells; and b, fluorescent field of the cultured Hec-1a cells following FAM-labeled siRNA transfection. (B) EZH2 knockdown was verified by western blot analysis. Growth curve of (C) Hec-1a and (D) Ishikawa cells after EZH2 siRNA transfection. Following transfection for 120 h, cell growth was significantly inhibited in both types of cells. ^*^P<0.05 and ^**^P<0.01. EZH2, enhancer of zeste homolog; CCK-8, Cell Counting Kit-8.

To evaluate the effect of EZH2 on cell proliferation, knockdown of EZH2 was performed in endometrial cancer cells, Hec-1a and Ishikawa, by siRNA. Subsequently, cell viability was analyzed using the CCK-8 assay. Scrambled siRNA, labeled by FAM, was transfected at the same time to observe the transfection efficiency (Fig. 2A), and the knockdown effect on EZH2 was validated by western blotting (Fig. 2B). The inhibition effect started 48 to 96 h after cell transfection and was significantly inhibited in Hec-1a and Ishikawa cells at 120h (Fig. 2C and D). Cell growth was inhibited after EZH2 knockdown in endometrial cancer cells.

## Discussion

The widely used World Health Organization system classifies endometrial hyperplasia into four levels according to glandular crowing and nuclear appearance: Simple, complex, simple atypical and complex atypical hyperplasia (17). Simple hyperplasia refers to diffuse and variably sized glands with a normal ratio of glands to stroma; complex hyperplasia consists of architecturally irregular glands and an increased gland-to-stroma ratio. When there is nuclear enlargement with chromatin evenly dispersed or clumped, it is called simple or complex atypical hyperplasia. In the present study, normal endometrium and simple hyperplasia did not show significantly elevated expression of EZH2, while complex hyperplasia displayed significantly increased expression of EZH2. Furthermore, high EZH2 expression also presented in the majority of atypical hyperplasia and endometrial cancer samples. EZH2 starts to become expressed in the precursor lesions of endometrial cancer, which indicates that high EZH2 expression is an early event of endometrial cancer carcinogenesis.

The identification of biological markers in normal and hyperplastic endometrium that reliably predict an increased risk of progression to endometrial cancer would provide important clinical benefits. The long-term risk among women with simple or complex hyperplasia is <5%, but the risk among women with atypical hyperplasia is ~30% (18). The results of the present study showed that there was a significant difference in endometrial EZH2 levels between normal subjects and patients with complex hyperplasia. This implicates that endometrial EZH2 expression may be used as a screening approach to identifying high-risk subpopulation with a potential to progress to carcinoma. Current WHO classification of endometrial hyperplasia is problematic due to poor diagnostic reproducibility. The significant differences in epithelial expression of EZH2 may provide clues to identify simple or complex hyperplasia.

Moreover, the present study demonstrated that EZH2 overexpression was associated with myometrial invasion in endometrial cancer. The high level expression of EZH2 (IHC score, 2 or 3) was observed in 100% of cases with deep (≥1/2) myometrial infiltration, while no patients with low EZH2 expression exhibited deep myometrial infiltration. This indicates that endometrial cancers with high expression levels of EZH2 tend to be more invasive.

EZH2 has been reported to be involved in the regulation of invasion-related factors, including E-cadherin, β-catenin and MMP9 (19). By silencing EZH2, the mRNA expression levels of E-cadherin and Keratin 18 increased by 177 and 158%, respectively; while mesenchymal markers, β-catenin and N-cadherin, decreased by 18.04 and 41.18%, respectively, in nasopharyngeal carcinoma cells (20). However, overexpression of EZH2 was correlated with reduced expression of E-cadherin, which led to reduced cell migration and invasion (21). Moreover, knocking down EZH2 expression suppresses the cell invasion by downregulating E2F1 and MMP9 in endometrial (22) and in colorectal (23) cancer. In the present study, the finding that EZH2 was associated with deep myometrial invasion supports the hypothesis that EZH2 may be an important factor in the invasion of endometrial cancer cells by regulating E-cadherin, β-catenin and MMP9.

The depth of myometrial invasion is an important factor in prognosis, determination of clinical stage or surgical procedure selection. The differential expression of EZH2 in stage IA, IB and IC endometrial cancer was not significantly different and indicated that it is more important in the discrimination of early-stage endometrial cancer than advanced endometrial cancer (stage II, III and IV).

The biological function of EZH2 in endometrial cancer was further evaluated. Reduced cell proliferation was identified after EZH2 was silenced by siRNA, indicating that EZH2 was involved in the cell proliferation of endometrial cancer. It has been reported that EZH2 expression is positively correlated with expression of Ki-67 (24). Ki-67, a marker of cell proliferation, is involved in cell mitosis and is positively correlated with the number of cells that are about to enter mitotic phase. Overexpression of Ki-67 is often observed in malignant tumors and can be a reliable marker of enhanced proliferation. Although no correlation was identified between the expression of EZH2 and Ki-67 in the present study, this may have been due to the insufficient sample size.

In the present study, high EZH2 expression appeared to be associated with LVSI. High expression of EZH2 was seen in 56% (15/27) of cases without LVSI, while it was observed in all (6/6) cases with LVSI. However, a significant positive correlation between the overexpression of EZH2, focal adhesion kinase (FAK) and phosphorylated FAK, as wells as angiolymphatic invasion and lymph node metastasis in endometrial cancer were identified by Zhou *et al* (25), suggesting that EZH2 may regulate endometrial cancer migration along with FAK through modulating E-cadherin.

In conclusion, overexpression of EZH2 correlates with deep myometrial invasion, LVSI and enhanced cell proliferation of endometrial cancer cells. It may predict a more aggressive biological behavior in endometrial carcinoma and may serve as a potential therapeutic target for the treatment of endometrial cancer. Future studies are required to further evaluate the biological function of EZH2 in endometrial tissue and prospectively assess its potential role as a prognostic marker.

## Figures and Tables

**Table I:** The relationship between expression of EZH2 and clinicopathological characteristics in endometrial cancer.

	EZH2 expression, n		
		
	Score=0/1	Score=2/3	P-value
Case no.	12	21	
Age, years			
<60	10	11	0.133
≥60	2	10	
Tumor grade			
G1	5	10	0.694
G2	3	2	
G3	2	5	
Type 2	2	4	
FIGO stage			
I	11	19	1.000
II, III and IV	1	2	
Depth of myometrial invasion			
Limited to endometrium	2	0	**0.013**
<1/2	10	14	
≥1/2	0	7	
LVSI			
No	12	15	*0.065*
Yes	0	6	
Nodal metastasis			
Negative	10	17	0.133
Positive	2	4	
Estrogen receptor			
Negative	2	3	1.000
Positive	10	18	
Progesterone receptor			
Negative	1	3	1.000
Positive	11	18	
P53			
Negative	9	15	1.000
Positive	3	6	
Ki-67			
<10% positive	0	3	0.573
10–39% positive	4	6	
≥40% positive	8	12	

a P-value <0.05 in bold, P-value between 0.05 and 0.10 in italic.
